# Kawasaki disease in children and adolescents: clinical data of Kawasaki patients in a western region (Tyrol) of Austria from 2003–2012

**DOI:** 10.1186/1546-0096-12-37

**Published:** 2014-09-02

**Authors:** Elisabeth Binder, Elke Griesmaier, Thomas Giner, Michaela Sailer-Höck, Juergen Brunner

**Affiliations:** 1 Clinic of Pediatrics I, Department of Pediatrics, Medical University Innsbruck, Anichstrasse 35, 6020 Innsbruck, Austria; 2 Clinic of Pediatrics II, Department of Pediatrics, Medical University Innsbruck, Anichstrasse 35, 6020 Innsbruck, Austria

**Keywords:** Kawasaki disease, Clinical symptoms, Fever duration, Age distribution, Coronary complications

## Abstract

**Background:**

Kawasaki disease (KD) is a rare vasculitis seen predominantly in children. In developing countries, it is the leading cause of childhood-acquired heart disease. Besides a case report from 1981 there have been no data published dealing with the epidemiology and clinical aspects of KD in Austria.

**Methods:**

The purpose of the present study was to investigate the clinical spectrum of KD in a geographically determined cohort of infants, children, and adolescents that were diagnosed and treated at the University Hospital of Innsbruck from 2003–2012.

**Results:**

Thirty-two patients were included in the study with a median age of 32.96 months (2–192). 59.4% of the patients were aged between six months and four years. The male-to-female ratio was 1:1.13. Clinical examination revealed non-purulent conjunctivitis and exanthema as the most common symptoms (84.4%). 75% showed oropharyngeal changes, 21.9% had gastrointestinal complaints such as diarrhoe, stomachache or vomiting prior to diagnosis. One third of the patients were admitted with a preliminary diagnosis, whereas 78.1% were pre-treated with antibiotics. The median fever duration at the time of presentation was estimated with 4.96 days (1–14), at time of diagnosis 6.76 days (3–15).75% were diagnosed with complete KD, and 25% with an incomplete form of the disease. There was no significant difference in the duration of fever neither between complete and incomplete KD, nor between the different age groups. Typical laboratory findings included increased C-reactive protein (CRP) (80.6%) and erythrocyte sedimentation rate (ESR) (96%),leukocytosis (48.4%) and thrombocytosis (40.6%) without any significant quantitative difference between complete and incomplete KD. Coronary complications could be observed in six patients: one with a coronary aneurysm and five with tubular dilatation of the coronary arteries. Our patient cohort represents the age distribution as described in literature and emphasizes that KD could affect persons of any age. The frequency of occurrence of the clinical symptoms differs from previous reports – in our study, we predominantly observed non-purulent conjunctivitis and exanthema.

**Conclusion:**

KD should always be considered as a differential diagnosis in a child with fever of unknown origin, as treatment can significantly decrease the frequency of coronary complications.

**Electronic supplementary material:**

The online version of this article (doi:10.1186/1546-0096-12-37) contains supplementary material, which is available to authorized users.

## Background

Kawasaki disease (KD) is the second most common vasculitis in children. Clinically, KD is characterized by prolonged fever of at least five days of duration and certain clinical features, occurring often sequentially in a non-predefined order [1]. Coronary artery aneurysms as a sequelae of vasculitis of KD occur in 20–25% of untreated children. In developing countries, KD has replaced acute rheumatic fever as the leading cause of acquired heart disease in children [2, 3]. The classic diagnosis of KD is based on fever for more than five days associated with at least four of the five principle features such as such as polymorphic exanthema, changes of the oropharynx, bilateral non-purulent conjunctivitis, cervical lymphadenopathy and changes to the extremities such as erythema [4]. Besides, some patients do not meet the classic criteria, showing only some of the principal features, thus frequently causing a diagnostic challenge. The term “incomplete KD” describes KD in children presenting the characteristic fever but less than four classical diagnostic criteria [5, 6]. “Atypical KD” is defined as KD with any unusual presentation [7] and occurs more often in infants [8]. Their young age and the potential for not receiving timely treatment puts them at higher risk of developing coronary artery lesions [8]. It is also broadly agreed that KD can be diagnosed in the absence of full criteria when coronary abnormalities are present [9].

Over 80% occur between the ages of six months and four years [10]. The low-incidence in the first six months suggests that most infants are protected by acquired maternal antibodies. The occurrence of KD is described 1.5 times more common in males than in females [11–13]. The predominant season for KD varies in different countries. In Europe seasonal peaks are described in winter and spring [14–16].

KD now occurs in limited regional epidemics [17]. Although, it has been reported in most ethnic groups, the variation of incidence between countries is striking [14, 16, 17]. It is overrepresented among the Asian population with an incidence rate in Japan estimated between 120 and 200 cases per 100000 children younger than five years whereas the incidence in the United States and in several European countries is reported of 4 to 20 per 100000 children less than five years [14]. The incidence remains high in those migrating to lower incidence countries [16]. In general, the incidence of KD appears to be increasing in a number of countries [11]. In siblings of affected children it is 10 to 15-fold higher than the population incidence [18] and familial occurrence is described [19]. These observations suggest that disease susceptibility may be influenced by genetic and cultural factors [20].

The present study was designed to investigate the clinical spectrum of KD in an entire geographically determined cohort of pediatric patients.

## Methods

### Data collection

Our investigation is a retrospective study including all Kawasaki patients, diagnosed and treated between 2003 and 2012 at Innsbruck Medical University (Tyrol, Austria). This clinic serves a catchment area including the entire province of Tyrol. Referrals of Kawasaki patients from other regional hospitals are frequent. The data was collected from patient reports and physician remarks. The presented research is in compliance with the Helsinki Declaration.

### Statistical analysis

Statistical analyses were performed using SPSS software version 20.0 for Windows (SPSS Inc. Chicago, IL, USA). Clinical and patient data (age, gender, duration of fever until diagnosis or primary presentation, number of patients with cardiac complications, WBC and platelet counts, CRP levels) are expressed as median ± range or mean ± standard deviation (SD). Data distribution was evaluated by means of histogram analysis and realization of a Shapiro-Wilk test. For comparison of different groups, the unpaired *T*-test or Mann–Whitney *U*-test was applied when appropriate. Results were regarded as statistically significant when p < 0.05.

## Results

### Participants

During the survey period 32 children (n = 32) were diagnosed with KD at the University Hospital of Innsbruck. Based on the clinical based definitions of KD 75% of patients were diagnosed with complete, 25% with incomplete KD. Based on the observation that over 80% occur between the age of six months and four years, we categorized our patient cohort into three age groups: younger than six months, six months to four years, and older than four years. 59.4% of our patients were in the group of six months to four years, whereas 18.8% were younger than six months and 21.9% older than four years. The median age of the cohort was 25 months (2.08 years), ranging from 2 months to 16 years. The male-to-female ratio was 15 vs. 17. 59.4% of the patients were diagnosed in winter and spring.

### Clinical aspects

31.3% of our patients were transferred from a general practitioner, pediatrician, or another hospital with a different primary diagnosis such as sepsis, scarlet fever, exanthema subitum or viral exanthema, superinfection of a dermatitis, urinary tract-infection, tonsillitis, otitis media and aphthous stomatitis. In three of our patients HHV6 could be confirmed whereas one of them suffered from a Norovirus infection in addition to KD. 78.1% of our patients were already treated with antibiotics prior to admission to our outpatient clinic.

**Figure 1:**
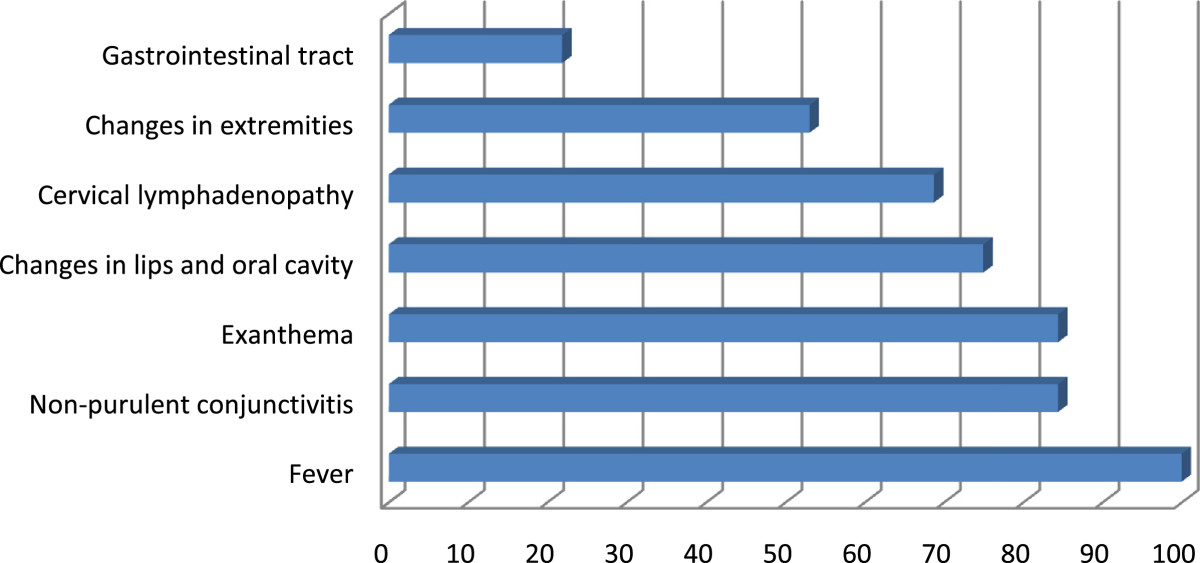
**Appearance rate of symptoms of patients diagnosed with KD.**

For 29 of 32 cases the duration of fever could be verified by the patient’s report. The mean duration of fever at primary consultation was 4.95 days (+/-2.97) with one patient reporting a history of prolonged fever two weeks despite antibiotic treatment. The mean time of diagnosis was 6.89 days (+/-3.38) after the fever had started, including two patients whom were not diagnosed before day 15. There was neither significant difference in the duration of fever until diagnosis between complete and incomplete KD, nor a significant difference of the duration of fever among the different age-groups.Clinical examination revealed that the most common symptoms were fever (100%), non-purulent conjunctivitis as well as an exanthema (both 84.4%). 75% of the patients showed changes in lips and oral cavity including enanthema (43.8%), red, cracked lips (46.9%) and strawberry-tongue (18.8%). Cervical lymphadenopathy was observed in 68.8% of all cases. 53.1% of our patients experienced changes in extremities like palmar or plantar erythema (Figure 1). 21.9% complained about gastrointestinal symptoms such as diarrhea, stomach ache or vomiting.

### Laboratory findings

In 81.3% (n = 26, 1 missing data) of our patients an elevated C-reactive protein (CRP) with a mean value of 11.83 mg/dl could be measured, 46.2% showing a CRP > 10 mg/dl. In 25 patients the erythrocyte sedimentation rate (ESR) was measured and showed a mean value of 88.08 mm/h (12–170 mm/h) after one hour and 114.82 mm/h (34–170 mm/h) after two hours – besides one patient all of them had a significant elevated ESR. About half of the patients (46.9%, 1 missing data) showed a leukocytosis and 40.6% (1 missing data) developed a thrombocytosis while they were hospitalized. There was no significant difference regarding laboratory findings (CRP, ESR and leukocytes) between complete and incomplete KD (p > 0.05).

### Cardiac complications

In total, six patients (18.75%, 1 missing data) had cardiac complications; five patients experienced tubular dilatation of the coronary arteries and one patient suffered an aneurysm. Pericardial effusion could be observed in one patient. The patients with tubular dilatation of the coronary arteries had fever lasting five to 15 days (mean duration of fever of those patients was 9 +/- 5.04 days).

In the present study in all patients with cardiac complications ESR was elevated. The patient with the aneurysm had an ESR of 170/170 mm/h, while the mean ESR-value of the patients with cardiac complications was 94 (±65.73)/127 (±41.47) mm/h and therefore just slightly higher than the mean value of 86.6 (+/-47.69)/111.23 mm (+/-47.06)/h of those without a complication (p > 0.05). The mean value of the CRP (9.91 mg/dl ±8.70) did not differ from that of the entire study population (9.98 mg/dl) (p > 0.05). With the exception of one patient for whom laboratory values were inconclusive, all of them had leukocytosis. Three, in addition, developed thrombocytosis while the disease took its course.

## Discussion

This retrospective analysis on KD is the first study of its kind in Austria. One of the most interesting findings was that almost one third of patients were transferred to our hospital with another preliminary diagnosis indicating that KD is often hidden by fever of unknown origin. However, the average length of time until the beginning of adequate treatment was only 6.75 days.

The number of the patients is comparable with certain epidemiologic studies on KD done in other European countries such as Italy [21], Iceland [22], northern European countries [23] and the Netherlands [24]. Our patients showed a balanced male-to-female ratio, which is different to other studies that usually reveal the predominance of KD in boys [14, 21, 23]. This might be explained because of the rather low number of patients included in this study, however it represents an entire cohort of children and adolescents of one region.

According to previous studies, over 80% occur between the ages of six months and four years [10] whereas a recently conducted study in northern Europe revealed only 67,8% of Kawasaki patients younger than 5 years [23]. In our patient cohort, 59.4% were at the age between six months and four years and therefore meet the most common age distribution. The low incidence in the first six months suggests that most infants are protected by acquired maternal antibodies [2]. One patient was diagnosed with incomplete KD at the age of 192 months, whereas all the others were younger than 69 months. This patient was treated successfully with intravenous immunoglobin (IVIG) and aspirin and did not suffer from any complications.

Clinical features like a rash, non-purulent conjunctivitis, oropharyngeal changes, lymphoadenopathy and changes to the extremities are known in KD and often appear sequentially in a non-predefined order [1]. In contrast, in our study up to 85% of patients experienced a non-purulent conjunctivitis and an exanthema. Cervical lymphadenopathy was observed in 68.8%.

Multiple non-cardiac clinical findings may also be observed in patients with KD including arthritis or arthralgia [21]. Several of our patients presented with arthralgia, one suffered from painful gonarthritis and coxitis. Those accompanying, unspecific symptoms often result in diagnoses other than KD, especially if the patient presents with the incomplete or atypical type of the illness and therefore often postpone adequate treatment. Other unspecific symptoms that may impede prompt diagnosis are gastrointestinal complaints. Diarrhea, vomiting, and abdominal pain occur in approximately one-third of Kawasaki patients [9]. Even if gastrointestinal symptoms are seen often, mesenteric arteritis is found rarely [24]. Reports of segmental thickening of the small-bowel wall in the abdominal ultrasound of KD patients are published [25]. The changes are thought to result from bowel-wall edema due to vasculitis of the supplying vessels and might therefore be used as an additional diagnostic sign in KD. Exactly 21.9% of our patients suffered from gastrointestinal symptoms whilst gallbladder hydrops could be observed only in three of our patients.

Certain common pitfalls in the diagnosis of KD are bacterial lymphadenitis, urinary tract infection, viral meningitis, sepsis, and occasionally a child may present acute abdomen [26]. Almost one-third of our patients were transferred with a preliminary diagnosis, and even 78.1% were already treated with, sometimes several different, antibiotics because of fever for one or two days despite a suspected diagnosis for which an antibiotic treatment would be recommended.

Many studies were done to predict the risk of cardiac involvement in KD [27–29]. In addition to young age [8], duration of fever (presumably reflecting the severity of ongoing vasculitis) has been confirmed as a powerful predictor of coronary artery aneurysms in various studies [30]. Reflecting the duration of fever until diagnosis it could be observed that even six patients were diagnosed with KD before day five. As the data was collected from reports and remarks a bias could be the reason for this observation. Otherwise it prompts the following as previously investigated question: -can a diagnosis be made before day five if all other criteria are fulfilled and could an early IVIG-treatment prevent complications [31]. None of our patients treated before day five suffered from any cardiac complications. However, because of the small cohort this observation could only be a coincidence. In comparison to an Italian study that shows how higher values of CRP may play a crucial role in determining the development of coronary complications [32], our analysis revealed no laboratory value or any distinct clinical symptom that can be correlated with the development of coronary aneurysms. An echocardiography is therefore necessary whenever KD is suspected.

The maximum ESR, the extent of proinflammatory cytokine production and the degree of neutrophil activation have been shown to increase the risk of coronary damage [1]. An investigation in Taiwan showed that the effects of neutrophil count, dosage of IVIG treatment, and platelet count (<400 000) in acute coronary artery lesions in KD are important whereas age, dose of IVIG treatment and band count are related to the persistence of coronary lesions in chronic stage [27, 33]. Besides the dose of IVIG also the differences in the manufacturing process such as purification, preservation and the IgA content may influence the effectiveness of prevention of coronary complications [34]. Plasma levels of follistatin-like protein 1 (FSTL-1), a proinflammatory protein produced by mesenchymal tissue including cardiac myocytes was shown to be elevated in patients with acute KD and may correlate with the development of coronary artery aneurysms [35].

Besides leukocytosis, an elevation of ESR, increased CRP and thrombocytosis [36] plasma lipids are markedly altered in acute KD, with depressed plasma cholesterol, high-density lipoprotein (HDL), and apolipoprotein AI [4]. Hypoalbuminemia is common and urine analysis reveals intermittent sterile pyuria in about 33% of patients. In 2004, the AHA guidelines recommended measuring ESR, values of CRP, white blood cell count in blood and urine, platelet count, albumin, hemoglobin, and ALT to support the diagnosis in KD [9]. Our patients showed laboratory values characteristic for KD, however we could not observe any correlation between laboratory values and the course of the disease. In conclusion, the diagnosis of KD remains a clinical one- due to the fact that there is no specific test and the laboratory findings could just support the clinical suspicion. KD always has to be considered in a child, adolescent or even adult with fever of unknown origin.

## Conclusion

In conclusion, the diagnosis of KD remains a clinical one- due to the fact that there is no specific test and the laboratory findings could just support the clinical suspicion. KD always has to be considered in an infant, child and adolescent with a fever of unknown origin.

## Authors’ original submitted files for images

Below are the links to the authors’ original submitted files for images. Authors’ original file for figure 1
