# Supplementary figures and images for: Kawasaki disease in children and adolescents: clinical data of Kawasaki patients in a western region (Tyrol) of Austria from 2003–2012
Source: Pediatr Rheumatol Online J. 2014 Sep 2;12:37. doi: 10.1186/1546-0096-12-37 (PMC5350606; doi:10.1186/1546-0096-12-37)

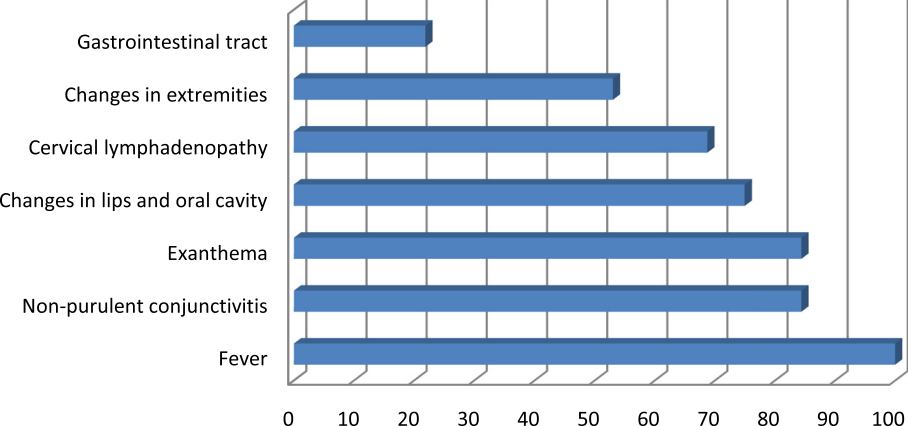

Supplement: Supplementary file 1 — Authors’ original file for figure 1 [file 12969_2014_1742_MOESM1_ESM.pdf]
